# Anterior Cruciate Ligament Reconstructed Patients Who Recovered Normal Postural Control Have Dissimilar Brain Activation Patterns Compared to Healthy Controls

**DOI:** 10.3390/biology11010119

**Published:** 2022-01-12

**Authors:** Yong Woo An, Yangmi Kang, Hyung-Pil Jun, Eunwook Chang

**Affiliations:** 1 Department of Health and Human Sciences, Loyola Marymount University, Los Angeles, CA 90045, USA; yongwoo.an@lmu.edu; 2 Department of Kinesiology, New Mexico State University, Las Cruces, NM 88003, USA; yangmik@nmsu.edu; 3 Department of Physical Education, Dong-A University, Busan 03722, Korea; hjun@dau.ac.kr; 4 Department of Kinesiology, Inha University, Incheon 22212, Korea

**Keywords:** electroencephalography, balance, neuroplasticity, knee injury, sensory integration, proprioception

## Abstract

**Simple Summary:**

We report that patients with anterior cruciate ligament reconstruction have similar postural control but different cortical activation patterns in several regions of the brain when compared to healthy controls. This is significant because dissimilar cortical activation patterns indicate that neural adaptation in the brain is responsible for motor coordination, possibly due to altered proprioception, despite having a surgical reconstruction after an anterior cruciate ligament injury. Such neuroplasticity in ACLR patients may imply compensatory neural protective mechanisms in order to sustain postural control, which is a fundamental functional skill in daily activities. We believe that our findings will elucidate other researchers and clinicians about the effects of a peripheral joint injury on the brain’s function during postural control.

**Abstract:**

Postural control, which is a fundamental functional skill, reflects integration and coordination of sensory information. Damaged anterior cruciate ligament (ACL) may alter neural activation patterns in the brain, despite patients’ surgical reconstruction (ACLR). However, it is unknown whether ACLR patients with normal postural control have persistent neural adaptation in the brain. Therefore, we explored theta (4–8 Hz) and alpha-2 (10–12 Hz) oscillation bands at the prefrontal, premotor/supplementary motor, primary motor, somatosensory, and primary visual cortices, in which electrocortical activation is highly associated with goal-directed decision-making, preparation of movement, motor output, sensory input, and visual processing, respectively, during first 3 s of a single-leg stance at two different task complexities (stable/unstable) between ACLR patients and healthy controls. We observed that ACLR patients showed similar postural control ability to healthy controls, but dissimilar neural activation patterns in the brain. To conclude, we demonstrated that ACLR patients may rely on more neural sources on movement preparation in conjunction with sensory feedback during the early single-leg stance period relative to healthy controls to maintain postural control. This may be a compensatory protective mechanism to accommodate for the altered sensory inputs from the reconstructed knee and task complexity. Our study elucidates the strategically different brain activity utilized by ACLR patients to sustain postural control.

## 1. Introduction

Postural control is a fundamental functional skill in activities of daily living, and it reflects integration and coordination of sensory information from multiple sensory modalities, including sensorimotor, vestibular, and visual systems [1,2,3]. Therefore, neuromechanical decoupling or dissociation between musculoskeletal and one or more of multimodal sensory systems may lead to a failure in maintaining postural control [1]. One of the factors associated with postural control impairment is task complexity. A single-leg stance assessment on varied levels of deflection of the base of support is one of the most common methods to evaluate the postural control [4]. More challenging levels of support with greater deviations of the platform create an unpredictable base of support for movement in any direction while standing in an upright position. Such an unanticipated external stimulus to the body can interrupt sensory neural processing in the central nervous system (CNS), ultimately leading to postural control impairment [5,6].

Observations of brain function using neuroimaging techniques have allowed examination of the neuromechanical decoupling associated with postural control impairment and demonstrated altered neural activity in the several regions of the brain as task complexity is more challenging [1,7,8]. As the unstable condition may induce unanticipated movement of the platform, it is possible that inconsistent proprioceptive inputs from the base of support to the CNS interfere with neural processing in the brain to maintain balance [1,8]. Growing evidence has demonstrated that peripheral joint injuries (i.e., ankle or knee sprains) can also result in postural control impairments [9,10]. Damaged joint proprioceptive mechanoreceptors may convey inappropriate afferent sensory information to the CNS, which can lead to failure in signal processing and integration needed for maintaining postural control [6,11,12]. Interestingly, recent neuroimaging studies have demonstrated that an anterior cruciate ligament (ACL) injury may permanently change neural activation patterns in the brain even after those patients underwent a surgical reconstruction [13,14,15]. As an increased task complexity [1,7,8] and/or a history of peripheral joint injury [13,14,15] that result in altered neural activation at specific regions of the brain may increase the risk of postural control impairment, understanding neural activity patterns during postural control may provide critical insights about different postural control strategies between patients with an ACL reconstruction (ACLR) and healthy controls. Therefore, the aim of this study was to compare the ability of postural control and neural activation in the brain during a single-leg stance task at two different task complexities (i.e., stable/unstable) between the ACLR patients and healthy control individuals. We hypothesized that ACLR patients who are presumed to recover the ability of postural control after the surgical reconstruction and rehabilitation program will demonstrate similar postural control regardless task complexities. Furthermore, the more challenging task condition (unstable) will have increased electrocortical activation when compared to the relatively easier task condition (stable), but greater increased electrocortical activation power in ACLR patients than the healthy controls.

## 2. Materials and Methods

### 2.1. Experimental Design

This study was a quasi-experimental, repeated measures design with two groups. The independent variables were one between-group factor (group: anterior cruciate ligament reconstructed patients (ACLR), healthy controls (CONT)) and one within-subject factor (postural control stability condition: stable platform (SP), unstable platform (UP)). The dependent variables were an overall postural control stability and electrocortical activity during the first three-second of the single-leg stance. The sample size was calculated using an *a priori* power analysis based on a published electroencephalography (EEG) study for postural control [16]. As a result, the minimum sample size to detect a moderate effect size at a probability of 0.05 with 80% power required 13 participants for each group.

### 2.2. Participants

Fifteen ACLR patients (5 female; age range, 19–28 years old (23.13 ± 3.20 years); height, 172.55 ± 9.95 cm; mass, 76.02 ± 17.22 kg) and age-, sex-, and leg dominance-matched 15 healthy volunteers (5 female; age range, 19–28 years old (23.07 ± 3.45 years); height, 175.68 ± 11.58 cm; mass, 71.09 ± 11.31 kg) who were physically active at least three days per week were recruited from local community and university between 1 October 2017 and 31 March 2019 (Table 1). The ACLR patients who were diagnosed with a unilateral ACL rupture (confirmed with magnetic resonance imaging) from 1 January 2011 to February 28, 2018, underwent a surgical reconstruction at least 6 months prior to the testing (average 2.97 ± 2.28 years). All patients were also cleared to return to their pre-injury level of physical performance by their physician. The healthy controls had normal knee function without history of neuromusculoskeletal injuries. As limb dominance, which is defined as the leg employed to kick a ball [14], is highly associated with morphological structural differences between left and right hemispheres in the brain [17], only right leg dominant participants were recruited. All participants had no neurological problems or history of lower extremity injury in the past 6 months, which can limit the quality of electrocortical activity and postural control, respectively. All participants read and signed an informed consent form that was approved by the New Mexico State University’s Institutional Review Board.

### 2.3. Postural Control Assessment

**Figure 1:**
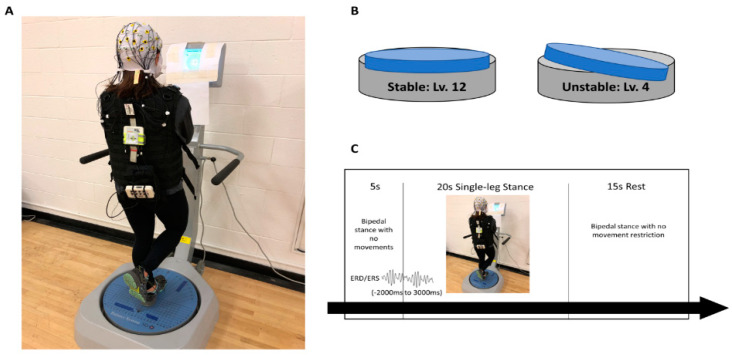
(**A**) Experimental setup. Visual feedback indicating participants’ center of pressure was presented in a display. (**B**) Postural control conditions: stable (level 12) and unstable (level 4). (**C**) Single trial of postural control experimental sequence.

Participants performed a single-leg stance task on both stable and unstable platforms (Balance System SD, Biodex, Shirley, New York, NY, USA) to examine postural control ability between ACLR patients and healthy controls. In the single-leg stance task, participants were asked to stand on the platform and were then instructed to maintain postural control on the investigator’s verbal cue while standing on the reconstructed limb for the ACLR group or the matched limb for the control group with 5° of knee flexion (Figure 1A). The contralateral non-stance limb was flexed at 45°, and both hands were crossed at contralateral shoulders. The foot location of the stance leg on the platform was determined prior to the postural control assessment that displays each participant’s center of gravity to ensure consistent foot position from session to session. The SP condition was chosen at level 12, which is defined as the zero degree of variation in any direction of platform movement, while the UP condition was chosen at level 4, which provides up to 15 degrees of platform movement in any direction during the stance period (Figure 1B).

Participants completed a practice session prior to each single-leg stance condition until they were familiarized with the tasks. Visual feedback, presented as a marker on a high-resolution color touch-screen LCD display (12.1 inches), was provided at eye level during the single-leg stance task (Figure 1A). The marker reflected participants’ resultant center of pressure in real time. Therefore, participants were instructed to constantly locate the marker as close to the center of the visual feedback display as possible, indicating better postural control. The order of conditions was randomly assigned for each participant, and each condition consisted of four blocks of five trials, lasting for 20 s. Participants had an appropriate rest period for 15 s and 5 min between trials and blocks, respectively, in order to minimize muscle fatigue (Figure 1C). If any failure trials during the single-leg stance occurred, i.e., loss of balance, hand off from the shoulder, or falling off the platform, participants ceased and repeated those trials. Twenty successful trials for each condition were included for data analysis, and an averaged overall stability index (OSI) of 20 trials was reported for the overall postural control stability score. The OSI is the average of displacement in degrees in all direction of platform movements. Thus, a greater OSI is indictive of poorer postural control.

### 2.4. Electrocortical Activity

The electrocortical activation data were conducted using a 32-channel mobile EEG system (LiveAmp, Brain Products, Munich, Germany) while standing on the platform (Balance System SD, Biodex, Shirley, New York, NY, USA). Thirty-two electrodes (Fp1, AFz, Fp2, F7, F3, Fz, F4, F8, FC5, FC1, FCz, FC2, FC6, T7, C3, Cz, C4, T8, CP5, CP1, CP2, CP6, TP9, P7, P3, Pz, P4, P8, TP10, O1, OZ, O2) were equally distributed across the entire scalp in compliance with the international 10:20 system. Electrodes ending with “z” align with the midline sagittal plane of the scalp. Electrodes ending with odd numbers are placed on the left hemisphere, whereas electrodes ending with even numbers are placed on the right hemisphere. Two electrodes at the mid-forehead (AFz and FCz) were used for a ground and reference, respectively. For EEG preparation, an appropriate size of electro-cap was placed on each participant’s head, and conductance electrolyte gel was inserted into each electrode. EEG signals that were ensured with sufficient electrode impedance (signal-to-noise ratio <5 kΩ) were recorded with a 500 Hz sampling rate using acquisition software (Brain Vision Recorder, Brain Products, Munich, Germany). A digital trigger produced from the balance system was used to synchronize the onset of each single-leg stance trial with electrocortical activity to capture real-time brain function. During the single-leg stance task, participants were instructed to keep their eyes focused on the target and were allowed to blink comfortably. They were told to minimize upper, lower, head, and facial muscle movements to reduce muscle artifacts. In order to acquire a baseline of non-task-related brain activity for data analysis, we also asked participants to stand with both feet on the platform without body movement for at least 5 s prior to the onset of each single-leg stance trial and then move from double limb support to the single-leg stance as quickly as possible on a verbal cue.

For electrocortical activity analysis, EEG data were analyzed using the Brain Vision Analyzer 2 software (Brain Products, Munich, Germany). Raw EEG data were first filtered (bandpass 0.1–40 Hz) and down-sampled to 256 Hz. A semiautomatic artifact rejection with visual inspection by an experienced investigator was used to review and exclude any raw EEG signals exceeding 50 µV/ms, maximal difference greater than 200 µV in 200 ms of intervals, or less than 0.5 µV in 100 ms of intervals, indicating non-brain-related electrical artifacts [18]. An Infomax Restricted Biased Independent Component Analysis algorithm was also used to detect and exclude ocular artifacts (i.e., blinking, vertical or horizontal eye movements). Cleaned and artifact-free EEG signals were then segmented into 5 s epochs (−2000 ms to 3000 ms, 1280 samples) for each participant and condition (Figure 1C). An averaged event-related desynchronization/synchronization (ERD/ERS), which is defined as decreased or increased percentage of power during the first 3 s of single-leg balance (0 ms to 3000 ms) relative to non-event related baseline brain activity (−2000 ms to 0 ms), retrospectively, was calculated in the theta (4–8 Hz) and alpha-2 (10–12 Hz) frequency bands for further analyses. Electrodes were selected for the centro-prefrontal (Fz), premotor/supplementary motor (FC1, FC2), primary motor (C3, Cz, C4), somatosensory (CP1, Pz, CP2), and primary visual (Oz) cortices, in which electrocortical activation is highly associated with goal-directed decision making [19], preparation of movement [20], motor output [21], sensory input [14], and visual processing [22], respectively. ERD/ERS values for the primary motor and somatosensory cortices were then separately analyzed into three subregions: central (electrodes endling with z), involved-limb (IL: contralateral electrodes endling with odd number for the right stance limb or even number for the left stance limb), and non-involved-limb (NIL: ipsilateral electrodes endling with odd number for the left stance limb or even number for the right stance limb).

### 2.5. Statistical Analysis

The Statistical Package for the Social Sciences version 25.0 (SPSS Inc., Chicago, IL, USA) was used for all statistical comparisons with a probability alpha level at 0.05. Any outliers that were greater than three standard deviations above or below than mean values were excluded from data analysis. Two-way repeated measures of ANOVAs with one between group factor (group: ACLR, CONT) and one within subject factor (condition: SP, UP) were used for the overall postural control scores and electrocortical activation (ERD/ERS) for each frequency band and electrodes. Mauchly’s test was used to evaluate the sphericity assumption. If the assumption of sphericity was violated (Mauchly’s test: *p* < 0.05), either the Greenhouse–Geisser correction (epsilon < 0.75) or Huynd–Feldt correction (epsilon > 0.75) of degrees of freedom was used to interpret statistical outcomes. The Bonferroni correction and pairwise multiple comparisons were applied for significant interactions. The partial eta squared (η^2^_p_) or Cohen’s *d* values were also reported to determine effect size. The partial eta squared values of 0.01, 0.06, and 0.14 and the Cohen’s d values of 0.2, 0.5, and 0.8 indicate small, moderate, and large effect sizes, respectively.

## 3. Results

### 3.1. Postural Control

**Figure 2:**
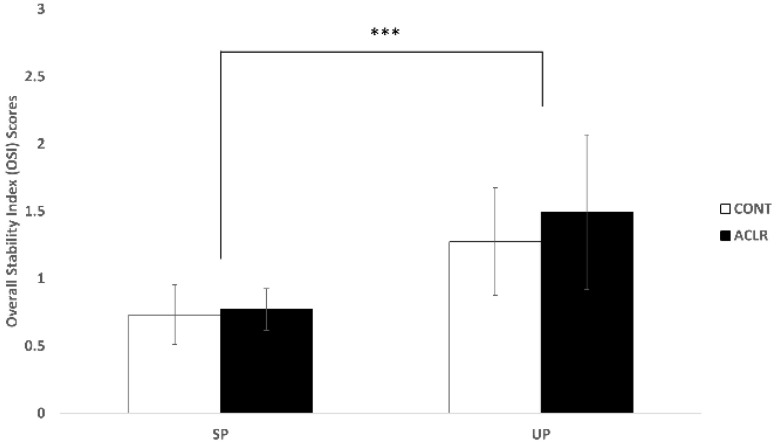
Postural control results. The unstable condition significantly increased the overall stability index scores when compared to the stable condition (*** *p* < 0.001).

There was a greater OSI score during the UP compared to the SP (*F*_(1, 25)_ = 50.443, *p* < 0.001, η^2^_p_ = 0.669). However, no significant group difference was found in OSI scores (*F*_(1, 25)_ = 1.280, *p* = 0.269, η^2^_p_ = 0.049) (Figure 2).

### 3.2. Electrocorticla Activation

**Figure 3:**
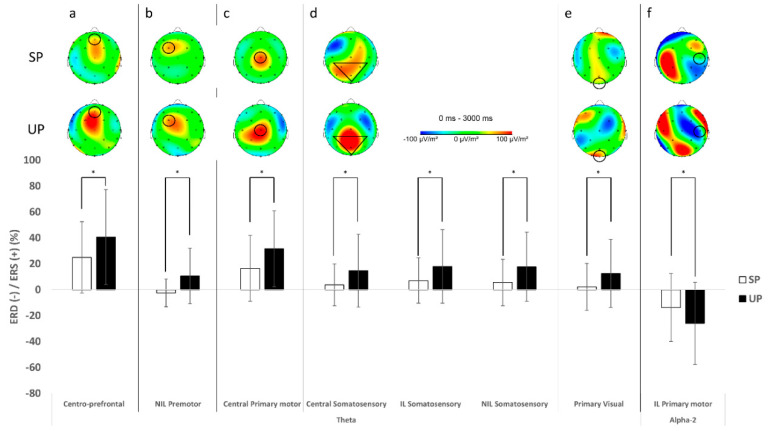
Mean topographical distribution (**top**) and plot (**bottom**) for significant main condition effects. The unstable condition significantly increased theta power in the centro-prefrontal (**a**), ipsilateral premotor (**b**), central primary motor (**c**), central and bilateral somatosensory (**d**), and primary visual cortices (**e**) and decreased alpha-2 power in the contralateral primary motor cortex (**f**), when compared to the stable condition (* *p* < 0.05).

**Figure 4:**
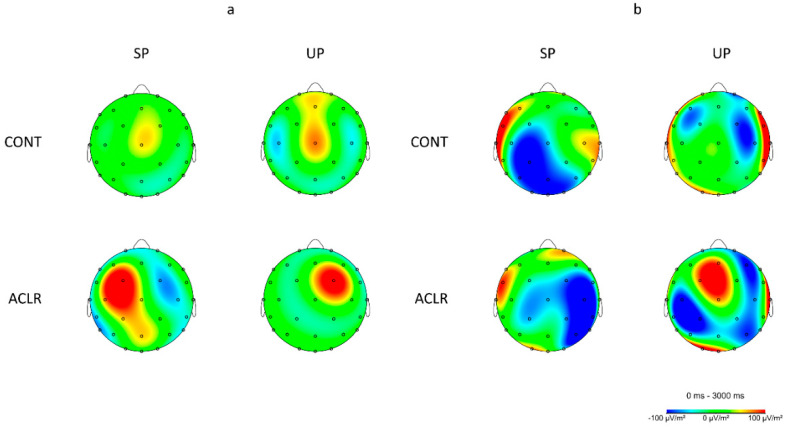
Mean topographical electrocortical activity distribution during a single-leg stance on the left limb for both groups; the right hemisphere is the contralateral cortices associated with the stance limb and the left hemisphere is the ipsilateral cortices associated with the non-stance limb. (**a**) Topographical distribution for theta frequency band (4–8 Hz) showing greater theta power at the contralateral premotor cortex in the ACLR group during the unstable condition than the control group as well as the stable condition. (**b**) Topographical distribution for alpha-2 frequency band (10–12 Hz) at the central prefrontal, ipsilateral primary motor, central and ipsilateral somatosensory, and primary visual cortices showing different neural activation patterns in the ACLR group when compared to the control group as well as between conditions.

Electrocortical activation results for theta and alpha-2 frequency bands at each electrode and condition are presented in Table 2. For the centro-prefrontal cortex (Fz), the UP condition increased more theta power (*F*_(1, 27)_ = 8.336, *p* = 0.008, η^2^_p_ = 0.236) when compared to the SP condition (Figure 3a). There was a significant group X condition interaction effect for alpha-2 power at the centro-prefrontal cortex (Fz) (*F*_(1, 25)_ = 8.317, *p* = 0.008, η^2^_p_ = 0.250; Figure 4b). Multiple pairwise comparisons showed that the ACLR group had greater increase in alpha-2 power during the UP condition when compared to the CONT group (*t*_(1, 28)_ = 2.093, *p* = 0.046, *d* = 0.76). Furthermore, the ACLR group had greater alpha-2 power during the UP condition than the SP condition (*t*_(1, 14)_ = 2.841, *p* = 0.016, *d* = 1.06).

The premotor/supplementary motor cortices (FC1, FC2) revealed a significant group X condition interaction effect for theta power at the IL (*F*_(1, 26)_ = 4.718, *p* = 0.039, η^2^_p_ = 0.154; Figure 4a). Multiple pairwise comparisons showed that the ACLR group had greater increase in theta power during the UP condition when compared to the CONT group (*t*_(1, 26)_ = 1.965, *p* = 0.060, *d* = 0.74). Furthermore, the ACLR group had greater theta power during the UP condition than the SP condition (*t*_(1, 13)_ = 1.909, *p* = 0.079, *d* = 0.69). There was also a significant condition main effect for the NIL with greater theta power during the UP condition than the SP condition (*F*_(1, 21)_ = 9.549, *p* = 0.006, η^2^_p_ = 0.313; Figure 3b). In alpha-2 frequency band, there was a significant main group effect for the NIL with a greater alpha-2 power in the ACLR group than the CONT group (*F*_(1, 24)_ = 5.347, *p* = 0.030, η^2^_p_ = 0.182) and no statistical significant condition main effect, but a slight trend toward greater alpha-2 during the SP than the UP (*F*_(1, 24)_ = 3.956, *p* = 0.058, η^2^_p_ = 0.142), while no significant differences between groups or conditions were found in the IL.

For the primary motor cortex (C3, Cz, C4), greater theta power was observed in the central region during the UP condition than the SP condition (*F*_(1, 25)_ = 8.159, *p* = 0.009, η^2^_p_ = 0.246; Figure 3c). Although no statistically significant group or condition main effects were observed for both the IL and NIL, the IL showed slight trends toward greater theta power in the ACLR group than the CONT group (*F*_(1, 26)_ = 3.541, *p* = 0.071, η^2^_p_ = 0.120) as well as during the UP condition than the SP condition for the NIL (*F*_(1, 25)_ = 3.731, *p* = 0.065, η^2^_p_ = 0.130). In alpha-2 frequency band, the IL showed a significant condition main effect with greater alpha-2 power during the SP than the UP (*F*_(1, 28)_ = 6.831, *p* = 0.014, η^2^_p_ = 0.196; Figure 3f), while the NIL revealed a significant group X condition interaction effect (*F*_(1, 24)_ = 7.535, *p* = 0.011, η^2^_p_ = 0.239; Figure 4b). Multiple pairwise comparisons showed that the ACLR group had greater increase in alpha-2 power during the SP condition when compared to the CONT group (*t*_(1, 22.054)_ = 2.382, *p* = 0.026, *d* = 0.86). Furthermore, the ACLR group had greater alpha-2 power during the SP condition than the UP condition (*t*_(1, 10)_ = 2.605, *p* = 0.026, *d* = 0.89).

For the somatosensory cortex (CP1, Pz, CP2), theta results revealed significant condition main effects for all three regions (central: *F*_(1, 28)_ = 5.557, *p* = 0.026, η^2^_p_ = 0.166, IL: *F*_(1, 27)_ = 6.335, *p* = 0.018, η^2^_p_ = 0.190, NIL: *F*_(1, 27)_ = 7.091, *p* = 0.013, η^2^_p_ = 0.208). There was an increase in theta power over the central, contralateral, and ipsilateral somatosensory cortexes during the UP when compared to the SP condition (Figure 3d). In alpha-2 frequency band, significant group X condition interaction effects were observed for the central (*F*_(1, 28)_ = 6.015, *p* = 0.021, η^2^_p_ = 0.177; Figure 4b) and the NIL regions (*F*_(1, 28)_ = 7.114, *p* = 0.013, η^2^_p_ = 0.203; Figure 4b). Multiple pairwise comparisons showed that the ACLR group had greater alpha-2 power during the SP than the UP in the central (*t*_(1, 14)_ = 2.166, *p* = 0.048, *d* = 0.51) and the NIL regions (*t*_(1, 14)_ = 1.953, *p* = 0.071, *d* = 0.47).

For the primary visual cortex (Oz), the UP condition also produced greater theta power (F_(1,27)_ = 5.061, *p* = 0.033, η^2^_p_ = 0.158) when compared to the SP condition (Figure 3e). There was a significant group X condition interaction effect for alpha-2 at the primary visual cortex (F_(1, 25)_ = 5.883, *p* = 0.023, η^2^_p_ = 0.191; Figure 4b). Multiple pairwise comparisons showed that the CONT group had greater alpha-2 power during the UP than the SP (t_(1, 13)_ = 2.174, *p* = 0.049, d = 0.51).

## 4. Discussion

The principle finding of this study was that the ACLR group who were presumed to recover the ability of maintaining postural control demonstrated different electrocortical activation patterns during the transition from double limb stance to single-leg stance when compared to healthy controls. Furthermore, the augmented cortical activation in several regions of the brain was observed when the single-leg stance task was more demanding.

### 4.1. ACLR Patients Had Similar Postural Control Patterns to Healthy Individuals

The OSI score was significantly increased when the base of support was more unstable during the single-leg stance, indicating poorer postural control when compared to the stable condition [1,7,23]. However, the ACLR group in this study showed similar overall postural control scores compared to the CONT group, regardless of conditions. As all ACLR patients were cleared to return to their pre-injury level of physical activities by their physicians at the time of the testing, this implies that the ACLR patients recuperated the ability of balance after the surgical reconstruction and rehabilitation process.

### 4.2. More Challenging Task Exhibited Altered Brain Activity during Balance Task

We further found that the unstable condition with a greater movement of the platform showed increased theta oscillation neural activity over the central prefrontal, ipsilateral premotor/supplementary motor, central and ipsilateral primary motor, central and bilateral somatosensory, and primary visual cortices, as well as less alpha-2 oscillation neural activity in the ipsilateral premotor/supplementary motor cortices, when compared to the stable condition. Theta power in the central prefrontal cortex is traditionally considered as neural excitability generated from the anterior cingulate cortex [24], which is highly associated with cognitive goal-directed decision-making processing of motor coordination [25]. A study by Sauseng et al. [26] demonstrated that a more challenging dual visuospatial executive condition increased theta power in both the frontal and parietal cortices when compared to a relatively easier cognitive condition. This simultaneously augmented theta power in the fronto-parietal network, known as the central executive system [26], which may reflect an integration of neural information from multi-modalities to appropriately coordinate motor responses in a rapid and complex environment [27]. Furthermore, Popivanov et al. [28] showed increased theta power in the premotor, supplementary motor, and parietal cortices prior to the onset of hand grip and suggested that this increase in theta power indicates preparatory muscle contraction. Similarly, Peterson and Ferris [7] showed increase in theta power in the anterior cingulate, parietal, and visual cortices when visual and physical demands became more challenging during waking and standing balance tasks. Further, alpha-2 oscillation neural activity indicates neural inhibition in the brain areas [29]. Therefore, less alpha-2 power in the ipsilateral premotor/supplementary motor cortices during unstable condition than stable condition implies more cortical activation in these cortices [29]. Our results were in agreement with previous studies demonstrating that more facilitated neural activation in these cortices reflect higher cognitive processing demands to cope with the level of task intensity by simultaneously detecting deviation of the platform in order to maintain balance [30].

### 4.3. ACLR Patients Had Dissimilar Brain Activity Patterns to Healthy Individuals during Early Single-Leg Stance

Growing evidence suggests that ACLR patients demonstrate similar postural control ability but different cortical activation patterns compared to healthy controls [31]. In early phase of the single-leg stance transitioning from the double limb supports, we found that the ACLR group had greater theta power in the contralateral primary motor cortex and greater alpha-2 power in the ipsilateral premotor cortex than the CONT group, regardless of conditions. Previous studies have demonstrated increased theta power in the primary motor cortex when the balance task became more difficult due to higher neural processing demand and optimized efferent outputs to maintain balance [16,32]. Although a lack of evidence still exists regarding the role of theta oscillation on the motor cortex [33], it has been suggested that oscillatory neural activity in theta frequency is accompanied with alpha-2 in neighboring cortices, such as premotor, supplementary, and parietal cortices [34]. Wheaton et al. [20] reported decreased alpha-2 power in the contralateral premotor cortex before the initial movement of the wrist extension and ankle dorsiflexion. As hemispheres regulate contralateral upper and lower extremities [35] and greater alpha-2 power indicates more inhibited neural activity in the area [36], the authors suggested that the decreased alpha-2 band in the contralateral premotor cortex prior to the wrist and ankle movements indicates increased cognitive processing needed for planning optimal muscle coordination. Given that no postural control difference was observed between groups in our study, the ACLR group had greater neural inhibitions in the ipsilateral premotor/supplementary cortices, corresponding to the non-injured limb, when compared to the CONT group. Although we have not examined the contribution of the non-injured, non-weight-bearing limb during the single-leg stance, attenuated neural activity in the ipsilateral premotor/supplementary cortices may be compensatory protective neural inhibition to focus on appropriate motor coordination of the reconstructed stance limb during early single-leg stance [37]. This implies that a peripheral joint injury, even though it is mechanically repaired by a surgical reconstruction, may permanently induce neural adaptation in the central nervous system, not only in the contralateral hemisphere responsible for the injured limb, but also in the ipsilateral hemisphere for the non-injured limb [38,39].

When the level of base of support stability was considered, the ACLR group had greater theta power in the contralateral premotor cortex and greater alpha-2 in the centro-prefrontal cortex during the unstable condition than the stable condition, as well as when compared to the CONT group. Previous research showed the increased theta power in the premotor cortex prior to the onset of hand grip, indicating successful preparatory muscle contraction [28], while increased alpha-2 in the centro-prefrontal cortex suggests more inhibited cognitive processing in a goal-oriented decision-making [26]. These may indicate that the ACLR group in the current study had greater neural activation for optimal preparation of muscles in the reconstruction limb while limiting cognitive processing in the prefrontal cortex as the balance task became more difficult.

Furthermore, the ACLR group had more decreased alpha-2 power in the central somatosensory cortex, while the CONT group showed more alpha-2 power in the primary visual cortex during the unstable condition than the stable condition. Decreased neural activity in alpha-2 oscillation in the somatosensory cortices indicates amplified sensory processing from the joints [13,15]. It is also known that alpha oscillation in the visual cortex decreases with an external visual feedback, but increases with more attentional cognitive processing [22]. These may suggest that the ACLR group relies on cortical activation in the somatosensory cortex responsible for processing sensory inputs from the lower extremity, while the CONT group is more dependent on internal cognitive processing to prepare and maintain balance [8,16]. Our results not only support the notion that the more challenging task demands additional cognitive processing but also suggest that the ACLR patients have different neural activation patterns following knee injury when compared to healthy controls. The different cortical activation patterns in ACLR group may be protective neural adaptations to optimize early preparation of dynamic postural control, particularly when the movement of base of the support becomes unpredictable.

There are several limitations in the current study. Although both ACLR patients and healthy controls were physically active at least three days per week by participating in their regular recreational activities, specific activity guidelines were not provided. Further, although we matched gender and sex between groups, we had an unequal number of male and female participants, which might affect their postural control ability after ACLR [40]. As different activity histories and/or routines among participants may affect their postural control ability, further research should consider recruiting participants from similar activity groups. Although post-operative postural control impairment in many ACLR patients might not be associated with knee function outcome measures [41], ACLR patients with better knee function tend to have more efficient brain function during postural control task [31]. All ACLR patients in the current study were cleared by their physicians for returning to pre-injury level of physical activity, but rehabilitation type and time after surgery and their knee functions were not quantified at the time of testing, which could have affected on postural control and electrocortical activation observed. Lastly, human movement during 20 s of postural control tasks was challengeable to preserve artifact-free EEG data, which resulted in 5 s windows of EEG data during the postural control tasks for EEG analysis. Further research should focus on minimizing human movement-related artifacts during EEG measures using more advanced EEG systems and analysis methods to observe brain activity during the whole 20 s of postural control tasks.

## 5. Conclusions

The effectiveness of postural control with brain activity was greatly influenced by the task complexity. More challenging single-leg stances with greater deflections of the platform movement resulted in poorer postural control in conjunction with significant alteration in brain activity during the early single-leg stance period, primarily in the cortical areas responsible for goal-directed decision making, preparation of movement, and sensorimotor and visual processing. Furthermore, patients with a history of surgical reconstruction after knee injury demonstrated similar postural control patterns but different brain activation patterns in those brain regions during early single-leg stance compared to healthy individuals, regardless of task difficulties. These results indicate that a peripheral joint injury may cause neural adaptation in the CNS such that neural processing strategies serve as compensatory protective mechanisms to accommodate for the task complexity. Our study provides insight into how ACLR patients utilize a different brain activity strategy to sustain postural control after a joint injury.

## Figures and Tables

**Table 1:** Participant demographic information.

Demographic Data (Mean ± SD)				
		CONT (N = 15)	ACLR (N = 15)	*p-*Value *
Sex, N	Male	10	10	
	Female	5	5	
Age, years		23.07 ± 3.45	23.13 ± 3.20	0.957
Height, cm		175.68 ± 11.58	172.55 ± 9.95	0.433
Weight, kg		71.09 ± 11.31	76.02 ± 17.22	0.362
Time from surgery, years			2.97 ± 2.28	

CONT, healthy controls; ACLR, anterior cruciate ligament reconstructed patients. * Reported from independent *t*-tests comparing group means.

**Table 2:** Significant different electrocortical activation patterns were observed between groups during the single-leg stance tasks.

		CONT		ACLR		Group-by-Condition Effect	
Hz	Cerebral Region	SP	UP	SP	UP	*F*	*p*
Theta	Central prefrontal ^c^	36.59 ± 28.47	44.33 ± 35.92	12.54 ± 20.74	36.67 ± 38.16	2.206	0.149
	IL premotor	12.09 ± 13.78	6.65 ± 15.57	2.56 ± 32.93	24.56 ± 31.47 *^a^*^b^	4.718	0.039 *
	NIL premotor ^c^	−2.31 ± 13.31	8.84 ± 16.23	−2.66 ± 7.42	12.59 ± 26.69	0.230	0.636
	Central primary motor ^c^	22.35 ± 28.35	30.40 ± 34.47	10.29 ± 21.19	32.98 ± 23.88	1.852	0.186
	IL primary motor	3.44 ± 14.97	5.34 ± 20.97	12.32 ± 19.50	21.38 ± 29.72	0.511	0.481
	NIL primary motor	2.71 ± 1055	11.52 ± 19.56	8.00 ± 17.59	19.23 ± 27.78	0.054	0.818
	Central somatosensory ^c^	1.67 ± 14.10	11.97 ± 30.24	5.83 ± 18.09	17.66 ± 26.29	0.027	0.871
	IL somatosensory ^c^	2.71 ± 12.75	12.86 ± 27.63	11.62 ± 21.03	23.47 ± 29.34	0.037	0.848
	NIL somatosensory ^c^	1.84 ± 11.97	11.83 ± 26.03	9.70 ± 22.48	23.91 ± 26.96	0.215	0.647
	Primary visual ^c^	−1.61 ± 14.55	8.73 ± 23.93	6.18 ± 21.00	16.68 ± 28.94	0.000	0.987
Alpha-2	Central prefrontal	5.57 ± 24.15	−2.37 ± 18.29	2.51 ± 8.67	15.14 ± 14.44 *^a^*^b^	8.317	0.008 *
	IL premotor	−11.16 ± 13.72	−12.33 ± 24.92	−10.02 ± 18.78	−3.02 ± 13.62	1.292	0.266
	NIL premotor ^d^	−7.92 ± 18.94	−14.32 ± 11.23	5.68 ± 23.00	−4.04 ± 9.40	0.169	0.684
	Central primary motor	−17.69 ± 23.53	−13.27 ± 26.00	−10.09 ± 22.55	−6.92 ± 23.23	0.026	0.873
	IL primary motor ^c^	−18.63 ± 23.36	−29.73 ± 30.56	−8.84 ± 28.84	−22.15 ± 33.48	0.056	0.814
	NIL primary motor	−22.47 ± 23.34	−19.09 ± 34.62	−5.67 ± 12.16 *^a^*^b^	−23.37 ± 25.23	7.535	0.011 *
	Central somatosensory	−25.65 ± 27.73	−17.28 ± 28.21	−13.23 ± 35.67 *^a^	−28.35 ± 21.69	6.015	0.021 *
	IL somatosensory	−23.08 ± 30.52	−23.73 ± 28.74	−11.58 ± 38.50	−26.30 ± 28.17	2.084	0.160
	NIL somatosensory	−26.49 ± 26.23	−14.84 ± 34.99	−8.75 ± 37.51	−24.34 ± 28.52	7.114	0.013 *
	Primary visual	−20.58 ± 23.43	−8.21 ± 25.43 *^a^	−6.49 ± 23.01	−14.12 ± 19.04	5.883	0.023 *

CONT: healthy controls, ACLR: anterior cruciate ligament reconstructed patients, SP: stable condition, UP: unstable condition, IL: contralateral cortical region associated with the stance limb, NIL: ipsilateral cortical region associated with the non-stance limb. * Significant group-by-condition interaction effect (*p* < 0.05), *^a^ significant condition difference from group-by-condition interaction effect (*p* < 0.05), *^b^ significant group difference from group-by-condition interaction effect (*p* < 0.05), ^c^ significant main condition difference (*p* < 0.05), ^d^ significant main group difference (*p* < 0.05).

## Data Availability

Data available on request due to restrictions eg privacy or ethical. The data presented in this study are available on request from the first author. The data are not publicly available due to their containing information that could compromise the privacy of research participants.
